# SH003 and Docetaxel Show Synergistic Anticancer Effects by Inhibiting EGFR Activation in Triple-Negative Breast Cancer

**DOI:** 10.1155/2022/3647900

**Published:** 2022-05-05

**Authors:** Yu-Jeong Choi, Kangwook Lee, Ji-Hye Yoon, Sung-Gook Cho, Yun-Gyeong Kim, Miso Jeong, Hyun-Ha Hwang, Seo Yeon Lee, Se-Eun Jung, Seong-Gyu Ko

**Affiliations:** ^1^Department of Science in Korean Medicine, Graduate School, Kyung Hee University, 26 Kyungheedae Rd., Dongdaemun-gu, Seoul 02447, Republic of Korea; ^2^Institute of Safety and Effectiveness Evaluation for Korean Medicine, 26 Kyungheedae Rd., Dongdaemun-gu, Seoul 02447, Republic of Korea; ^3^Department of Preventive Medicine, College of Korean Medicine, Kyung Hee University, 26 Kyungheedae Rd., Dongdaemun-gu, Seoul 02447, Republic of Korea; ^4^Department of Biotechnology, Korea National University of Transportation, 61 University Rd., Jeungpyeong, Chungbuk 27909, Republic of Korea

## Abstract

Although many anticancer drugs have been developed for triple-negative breast cancer (TNBC) treatment, there are no obvious therapies. Moreover, the combination of epidermal growth factor receptor- (EGFR-) targeted therapeutics and classical chemotherapeutic drugs has been assessed in clinical trials for TNBC treatment, but those are not yet approved. Our serial studies for newly developed herbal medicine named SH003 provide evidence of its broad effectiveness in various cancers, especially on TNBC. The current study demonstrates a synergic effect of combinatorial treatment of SH003 and docetaxel (DTX) by targeting EGFR activation. The combinatorial treatment reduced the viability of both BT-20 and MDA-MB-231 TNBC cells, displaying the synergism. The combination of SH003 and DTX also caused the synergistic effect on apoptosis. Mechanistically, the cotreatment of SH003 and DTX inhibited phosphorylation of EGFR and AKT in both BT-20 and MDA-MB-231 cells. Moreover, our xenograft mouse tumor growth assays showed the inhibitory effect of the combinatorial treatment with no effect on body weight. Our immunohistochemistry confirmed its inhibition of EGFR phosphorylation in vivo. Collectively, combinatorial treatment of SH003 and DTX has a synergistic anticancer effect at a relatively low concentration by targeting EGFR in TNBC, indicating safety and efficacy of SH003 as adjuvant combination therapy with docetaxel. Thus, it is worth testing the combinatorial effect in clinics for treating TNBC.

## 1. Introduction

Breast cancer is the leading cause of cancer-related death among women worldwide and has first surpassed lung cancer, with an estimated 2,261,419 new cases and 684,996 deaths in 2020 [1]. Breast cancer is commonly classified into three subtypes based on the expression status of estrogen receptor (ER), progesterone receptor (PR), and human epidermal growth factor receptor 2 (HER2). Among these subtypes, triple-negative breast cancer (TNBC) is characterized by an absence of ER and PR and a lack of HER2 overexpression. Although other breast cancer subtypes including ER+/PR+/HER2-, ER+/PR-/HER2-, and HER2 overexpression have been managed by targeted therapy such as endocrine therapy and anti-HER2 therapy with improved prognosis, there are no effective therapeutic ways for TNBC [2–4].

Epidermal growth factor receptor (EGFR) suffers autophosphorylation at multiple tyrosine residues via its interaction with its endogenous ligand and activates downstream signaling pathways including ERK, AKT, and STAT3 [5]. Therefore, EGFR-mediated events are multifaceted in the cells. EGFR is overexpressed in TNBC, thereby being proposed as a therapeutic target [6]. While BRCA mutation appears to be linked to EGFR overexpression in TNBC, underlying mechanisms are still unclear [7, 8]. Moreover, gain-of-function mutations of EGFR are not broadly detected in TNBC patients [9]. EGFR-targeting agents such as gefitinib, erlotinib, osimertinib, and cetuximab are often used in clinics. Although EGFR-targeting agents are currently approved for nonsmall cell lung cancer (NSCLC) on the basis of their effectiveness, the response is limited to patients with gain-of-function mutations of EGFR such as exon 19 deletion and L858R mutation [10]. Those agents are not working in NSCLC with wild-type EGFR overexpression, which is a reason that those agents fail in TNBC treatment even though molecular and cellular mechanisms are completely unclear [11–13]. Nevertheless, clinical trials evaluating the effect of EGFR-targeting agents in combination therapies with chemotherapies are still conducted for TNBC treatment [14].

Docetaxel (DTX), a semisynthetic analog of paclitaxel isolated from European yew (*Taxus baccata*), is one of chemotherapeutics that causes cell cycle arrest by interfering with microtubules functions [15, 16]. DTX was first approved for the treatment of cancer such as breast, ovarian, and nonsmall cell lung cancer, and especially used in treatment of TNBC or early stage of human breast cancers [17]. Even though DTX is the effective anticancer drug, most chemotherapeutic agents including DTX have faced to limitations such as drug resistance, cancer recurrence, and adverse effects [18, 19]. Therefore, it is urgent to enhance the effectiveness of DTX in TNBC treatment with no adverse effect. One of therapeutic ways is likely to reduce its dose, which could ameliorate adverse effects with keeping its effectiveness.

SH003 is an herbal mixture consisting three herbs *Astragalus membranaceus* (Am), *Angelica gigas* (Ag), and *Trichosanthes Kirilowii Maximowicz* (Tk), which has been developed as a novel anticancer drug against several cancers including prostate, cervical, pancreatic, and breast cancer [20–22]. Especially, SH003 inhibits the growth of MDA-MB-231 TNBC cell line by inducing apoptosis followed by autophagy, both *in vitro* and *in vivo* [23]. Moreover, a combination of SH003 and doxorubicin exhibits synergistic effect on TNBC [24]. In addition, SH003 sensitizes paclitaxel-resistant ER-positive MCF7 cells to paclitaxel by inhibiting p-glycoprotein (MDR1) activity [25]. Based on the chemotherapeutic effect of SH003 or DTX on TNBC, we hypothesized that SH003 treatment combined with DTX may be effective for treatment of TNBC.

The present study is aimed at evaluating the synergistic effect of SH003 and DTX in TNBC cells. We showed that cotreatment of SH003 and DTX synergistically enhances apoptotic cell death in BT-20 and MDA-MB-231 cells. Both *in vitro* and *in vivo* studies demonstrated that blocking EGFR signaling is a key in the inhibitory mechanism of combination therapy. These findings suggest that combination of SH003 and DTX would be one of beneficial therapeutic strategy for TNBC treatment.

## 2. Materials and Methods

### 2.1. SH003, Chemicals, and Reagents

SH003 was prepared as described in our previous study [26]. In brief, SH003 was provided from Hanpoong Pharm and Foods Company (Jeonju, Republic of Korea). Am, Ag, and TK were mixed at 1 : 1 : 1 ratio (*w*/*w*) and then extracted with 30% ethanol at 100°C for 3 h. Dried extracts were dissolved in 30% ethanol and stored at −80°C until use. DTX (Sigma-Aldrich, St. Louis, MO, USA) was dissolved in DMSO and stored at −20°C.

Thiazolyl blue tetrazolium bromide (MTT) powder was purchased from Sigma-Aldrich (St. Louis, MO, USA). FITC-conjugated Annexin V apoptosis Detection Kit and 7-aminoactinomycin D (7-AAD) were purchased from BD Pharmingen™ (BD Biosciences, San Jose, CA, USA) and Sigma-Aldrich (St. Louis, MO, USA), respectively. Anticleaved caspase-3, -PARP, -GAPDH, -p-EGFR (Tyr 1068), -p-EGFR (Tyr 1173), -EGFR, -p-AKT (Ser 473), -AKT, -p-C-Raf (Ser 338), -p-STAT3, and -STAT3 antibodies were purchased from Cell Signaling (Danvers, MA, USA). Anti-p-ERK and ERK antibodies were purchased from Santa Cruz Biotechnology (Santa Cruz, CA, USA). Anti-Ki67 and CD31 antibodies were purchased from Abcam (Cambridge, MA, USA). Horseradish peroxidase- (HRP-) conjugated secondary antibodies for mouse and rabbit were purchased from SeraCare Life Sciences (Milford, MA, USA).

### 2.2. Cell Culture

Human breast cancer BT-20 and MDA-MB-231 cell lines were purchased from Korean Cell Line Bank (Seoul, Republic of Korea) and cultured in DMEM or RPMI-1640 medium (WelGENE, Daegu, Republic of Korea) supplemented with 10% fetal bovine serum (JR Scientific, Inc., Woodland, CA, USA) and 1% penicillin/streptomycin solution (WelGENE, Daegu, Republic of Korea). The cells were maintained at 37°C in 5% CO_2_/95% air with 100% humidity.

### 2.3. MTT Assay and Combination Index Analysis

BT-20 and MDA-MB-231 cells were seeded in 96-well plates and then treated with SH003 (100, 300, and 500 *μ*g/mL), DTX (1, 10, 100, and 1000 nM), or combination. After 24 h incubation, cell viability was measured by MTT assay with an absorbance at 570 nm. The interaction (synergistic, additive, and antagonistic effects) between SH003 and DTX was assessed by combination index (CI) analysis using Compusyn software (ComboSyn, Inc., Paramus, NJ, USA) (http://www.combosyn.com). CI value was calculated by the equation as follows [27]:

(1) $$\begin{array}{c} CI_{x}=\frac{{\left(D\right)}_{1}}{{\left(D_{x}\right)}_{1}}+\frac{{\left(D\right)}_{2}}{{\left(D_{x}\right)}_{2}} \end{array}$$

(*D*_*x*_)_1_ is the dose of SH003 alone that inhibits _x_%. (*D*_*x*_)_2_ is the dose of DTX alone that inhibits _x_%. (*D*)_1_ the portion of SH003 in combination SH003 and DTX also inhibits _x_%. (*D*)_2_ is the portion of DTX in combination SH003 and DTX also inhibits _x_%. “*CI* < 1”, “*CI* = 1”, and “*CI* > 1” mean synergistic, additive, and antagonistic effect, respectively.

### 2.4. Apoptosis Analysis by Flow Cytometry

Apoptotic cell death was analyzed by Annexin V/7-AAD double staining. Cells were stained with Annexin V followed by staining with 7-AAD in the dark for 15 min at room temperature. Stained cells were detected by FACSCalibur (BD Biosciences, San Jose, CA, USA), and apoptotic cells were analyzed using CellQuest Pro version 5.2 (BD Biosciences, San Jose, CA, USA) software.

### 2.5. TUNEL Assay

DNA fragmentation was measured using TUNEL assay (Abcam, Cambridge, MA, USA), according to the manufacturer's protocol. After the treatment with SH003, DTX, or combination for 24 h, the cells were harvested and fixed in 4% paraformaldehyde (PFA) for 15 min at 4°C. The cells were washed with PBS, resuspended in 100 *μ*L of PBS, and then added 1 mL of 70% EtOH for 30 min on ice. Subsequently, the cells were washed twice in wash buffer and incubated with 50 *μ*L of DNA labeling solution containing TdT enzyme and Br-dUTP for 1 h at 37°C. The cells were added rinse buffer and centrifuged. Then, the samples were resuspended in 50 *μ*L of anti-BrdU-Red antibody solution for 30 min at room temperature in the dark. Stained cells were diluted with rinse buffer and analyzed in FL-2 channel by flow cytometry.

### 2.6. Transfection

Cells were seeded in 6-well plate and transfected with 1.5 *μ*g pCDNA3-Myr-HA-AKT2 (addgene #9016) plasmid using lipofectamine 3000 (Invitrogen, CA, USA). Cells were incubated in 10% FBS/antibiotic-free media for 24 h and then trypsinized and seeded for additional studies.

### 2.7. Western Blotting

Total protein was extracted with RIPA buffer containing 50 mM Tris-HCl (pH 7.5), 150 mM NaCl, 1% triton X-100, 2 mM EDTA, 0.1% SDS, and 1% sodium deoxycholate. Protein concentration was quantified by Bradford assay, and proteins were separated on 8-12% SDS-PAGE. Separated proteins were transferred to nitrocellulose membrane and blocked with 5% skim milk in PBS-T at room temperature for 1 h. The membrane was incubated with primary antibodies at 4°C overnight, washed, and incubated with horseradish peroxidase-conjugated secondary antibody for 1 h. Proteins were measured using the EZ-western detection kit (Dogen-Bio, Seoul, Republic of Korea).

### 2.8. *In Vivo* Studies

All animal studies were approved by Kyung Hee University Institutional Animal Care and Use Committee (KHU-IACUC). Five-week-old female BALB/c nude mice were purchased from Nara Biotech (Seoul, Republic of Korea). Mice were given to access to food and drinking water *ad libitum* and were housed in appropriate isolated cage under pathogen-free condition with 12 h light/12 h dark cycle at room temperature (22-25°C). To establish tumor xenograft mouse model, BT-20 cell suspension (1 × 10^7^ cells) in 100 *μ*L PBS was subcutaneously inoculated in the right flank of mice. Based on no observed adverse effect of levels (NOAEL) of SH003 from phase 1 clinical study (4,800 mg/day) and maximum tolerable dose of DTX (75 mg/m^2^) in cancer patients (NCT03081819) [28], animal equivalent dose was determined by following equation: Human equivalent dose (mg/kg) = Animal dose (mg/kg)∗(Weight_animal_ [kg]/Weight_human_ [kg])^(1 − 0.75)^ [29]. Human and mice weights are 65 kg and 0.02 kg, respectively. Based on Kleiber's law [30], exponent for body surface area is 0.75, which account for difference in metabolic rate. Mice were divided into four groups; control (*n* = 3), SH003 (*n* = 4), DTX (*n* = 4), and SH003 + DTX (*n* = 5). When tumor volume reached at 100 mm^3^, drugs were injected. DTX was administrated intravenously *via* tail vein once a week with 15.277 mg/kg, while DMSO to control or SH003 alone group. SH003 was orally treated three times a week with 557.569 mg/kg, while saline to control or DTX alone group. Body weights were measured three times a week, and tumor volumes were analyzed daily for 17 days. Tumor volume was calculated using the formula: width^2^ × length/2. The mice were euthanized, and tumors were isolated. Tumor tissues were fixed and embedded in paraffin. For hematoxylin and eosin (H&E) staining, tissues mounted on slide glasses were incubated with H&E solution. The immunohistochemical analysis was performed using Vectastain ABC-AP staining kit according to manufacturer's instruction (Vector Laboratories, Burlingame, CA USA). The slide was stained with Ki-67, CD31, p-EGFR (Tyr 1068), and cleaved caspase-3 after performing heat-induced antigen retrieval using sodium citrate buffer (pH 6.0). Images of H&E and IHC were obtained with microscope (Carl Zeiss, Germany) at a magnification of 40×.

### 2.9. Statistical Analysis

Data were shown as the mean and standard deviation from at least three experiments. The statistical differences of means among the groups were analyzed by one-way ANOVA followed by Dunnett's or Bonferroni's test. *P* value < 0.05 means statistically significant differences.

## 3. Results

### 3.1. Synergistic Effect of SH003 and DTX on TNBC Cell Viability

**Figure 1:**
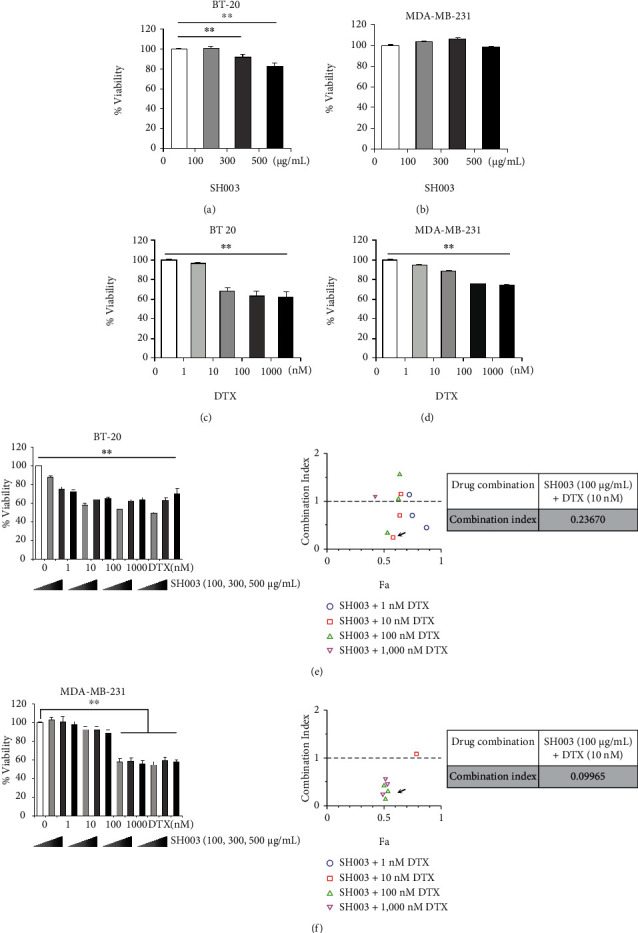
Combinatorial treatment of SH003 and DTX shows the synergic effect on TNBC cells. (a, b) BT-20 and MDA-MB-231 cells were treated with SH003 (100, 300, and 500 *μ*g/mL) for 24 h. Cell viability was measured by MTT assay. (c, d) Cells were treated with DTX (1, 10, 100, and 1000 nM) for 24 h and then MTT assay was performed. (e, f) Cells were cotreated with SH003 and DTX at the indicated doses for 24 h and analyzed by MTT assay. The combination index (CI) was calculated using CompuSyn software. *CI* < 1, *CI* = 1, and *CI* > 1 indicate synergistic, additive, and antagonistic effects, respectively. Also, Fa refers to inhibitory rate. Data represent three independent experiments. One-way ANOVA was used to compare the results, ∗*P* < 0.05, ∗∗*P* < 0.01 vs. control.

We first investigated the cytotoxic effect on TNBC cells by either SH003 or DTX. BT-20 and MDA-MB-231 cells were exposed to various concentrations of either SH003 or DTX for 24 h. SH003 did not affect the viabilities of both BT-20 and MDA-MB-231 cells, as the viabilities were about 83% and 98% at 500 *μ*g/mL dose, respectively (Figures 1(a) and 1(b)). However, DTX decreased the viabilities of both BT-20 and MDA-MB-231 cells in a dose-dependent manner (Figures 1(c) and 1(d)). Moreover, cotreatment of SH003 with DTX significantly inhibited the viability of TNBC cells, although not show a dose-dependent effect in cell survival (Figures 1(e) and 1(f)). To identify whether SH003 has the synergistic effect with DTX, the combination index (CI) value of drug pair was evaluated (Tables 1 and 2).

The highest synergistic effects in BT-20 and MDA-MB-231 cells were observed in combination of SH003 at 100 *μ*g/mL and DTX at 10 nM (CI: 0.24) and in combination of SH003 at 100 *μ*g/mL and DTX at 100 nM (CI: 0.1), respectively (Figures 1(e) and 1(f)). These findings indicated that SH003 and DTX have the synergistic effect at a relatively low concentration of DTX by blocking excessive toxicity accumulation.

### 3.2. Combination of SH003 and DTX Induces the Apoptotic Cell Death

**Figure 2:**
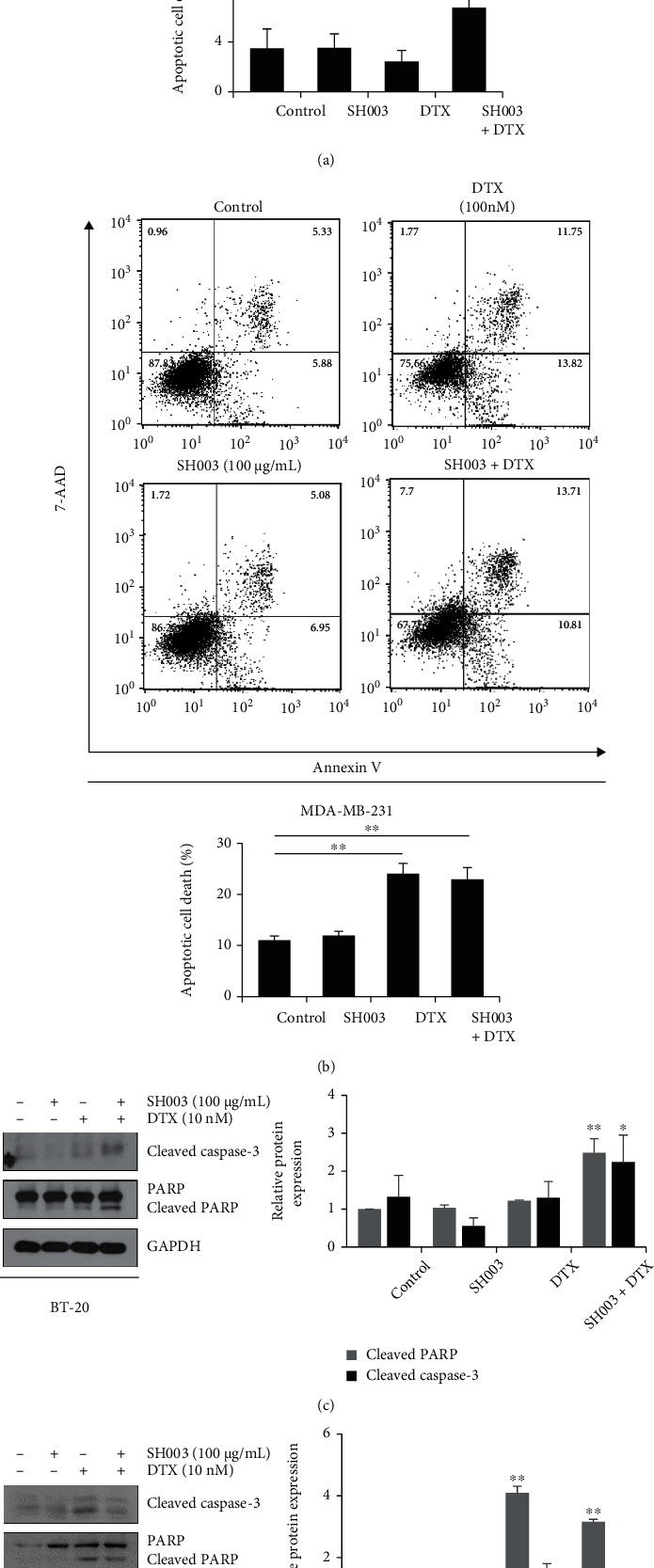
Combinatorial treatment of SH003 and DTX induces apoptotic cell death in TNBC cells. Cells were treated with SH003 (100 *μ*g/mL), DTX (10 nM or 100 nM), or combination for 24 h. (a, b) Apoptosis was analyzed by flow cytometry. The bar graph indicates apoptotic cells and data were shown as the mean ± SD on three independent experiments. (c, d) Protein expression levels were determined by western blotting. The relative expression of apoptosis-related proteins was quantified using Image J software and normalized to GAPDH. Data are means ± SD on three independent experiments. (e) Cells were treated with SH003 (100 *μ*g/mL), DTX (10 nM or 100 nM), or combination for 24 h. DNA fragmentation was detected using TUNEL assay and analyzed by flow cytometry. The bar graph shows the TUNEL positive cells. Data are expressed in the mean ± SD from three independent experiments and evaluated by one-way ANOVA, ∗*P* < 0.05, ∗∗*P* < 0.01 vs. control.

To examine apoptosis, BT-20 and MDA-MB-231 cells were treated with SH003 and/or DTX for 24 h, and then apoptosis was analyzed by flow cytometry. In Figures 2(a) and 2(b), the results showed that combined with SH003 and DTX induces apoptosis in both cells. In BT-20 cells, the combinatorial treatment enhanced the level of the early and late apoptotic cells, compared with SH003 or DTX alone (Figure 2(a)). Similarly, the combination of SH003 and DTX increased levels of cleaved PARP and cleaved caspase-3, which is known to marker of early apoptosis processes (Figure 2(c)). As shown in Figure 2(b), we observed that the cotreatment showed a similar increase in the early and late apoptotic cells (about 24%) with DTX (about 23%), while the increase in late apoptotic cells was higher in combinatorial treatment than in DTX. Also, apoptosis markers were induced by both DTX and cotreatment (Figure 2(d)). These results suggest that apoptosis process in MDA-MB-231 is distinct from BT-20. Therefore, we further performed the TUNEL assay to assess the induction of late apoptosis by combination treatment in MDA-MB-231 cells. The TUNEL assay can detect the DNA fragmentation, which is a representative marker occurred in end process of apoptosis [31, 32]. In BT-20 cells, SH003, DTX, and combination treatment increased the TUNEL positive cells, but there was no synergistic effect of combination treatment. On the other hand, the combination of SH003 and DTX caused an increase in TUNEL positive cells (about 10%) than DTX single (about 6%) in MDA-MB-231 cells (Figure 2(e)), indicating that combination treatment synergistically induces apoptotic cell death and especially is mediated by late apoptosis process in MDA-MB-231 cells and early/middle apoptosis in BT-20 cells. Taken together, these results demonstrated that combination treatment promotes the sensitivity of SH003 or DTX on TNBC cell by increasing the apoptosis.

### 3.3. SH003 Combined with DTX Induces Apoptosis through EGFR-AKT Signaling Pathway

**Figure 3:**
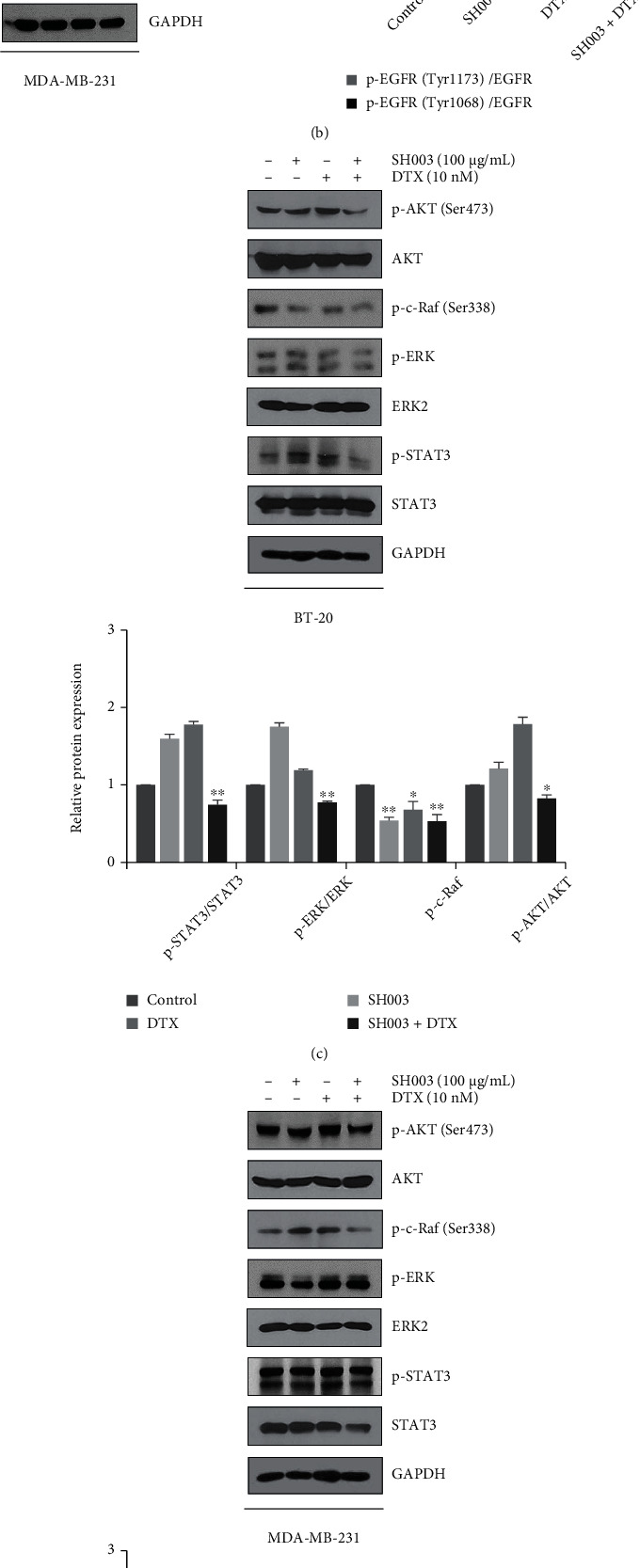
SH003 combined with DTX inhibits EGFR activation and EGFR downstream pathways. Cells were treated with SH003, DTX, or combination for 30 min. (a, b) Western blotting of EGFR in BT-20 and MDA-MB-231 cells. The bar graph indicates the relative protein expression and normalized to total EGFR. (c, d) Western blot analysis of EGFR downstream pathways in BT-20 and MDA-MB-231 cells. The relative expression of proteins was measured by Image J software. The data represent mean ± SD on three independent experiments. ∗*P* < 0.05, ∗∗*P* < 0.01 vs. control.

EGFR is overexpressed in TNBC cells [6], and EGFR-targeted therapeutics have been developed for treatment [33]. Therefore, we investigated if the combination of SH003 and DTX inhibits EGFR signaling pathway. As shown in Figures 3(a) and 3(b), combinatorial treatment of SH003 and DTX inhibited phosphorylation of EGFR in both BT-20 and MDA-MB-231 cells. AKT, Raf/ERK, and STAT3 are downstream signaling factors of EGFR-mediated signaling and known to activate tumor growth and metastasis [34]. Furthermore, many studies have been focused on AKT, ERK, and STAT3 as therapeutic targets in TNBC cells [35–37]. Therefore, we assessed whether combination of SH003 and DTX regulates downstream pathway of EGFR. Compared with SH003 or DTX alone, combinatorial treatment decreased phosphorylation of AKT in both BT-20 and MDA-MB-231 cells. However, the combinatorial treatment inhibited phosphorylation of Raf/ERK and STAT3 only in BT-20 cells (Figures 3(c) and 3(d)), suggesting that molecular module of EGFR-mediated downstream signaling may be different between BT-20 and MDA-MB-231 cells.

**Figure 4:**
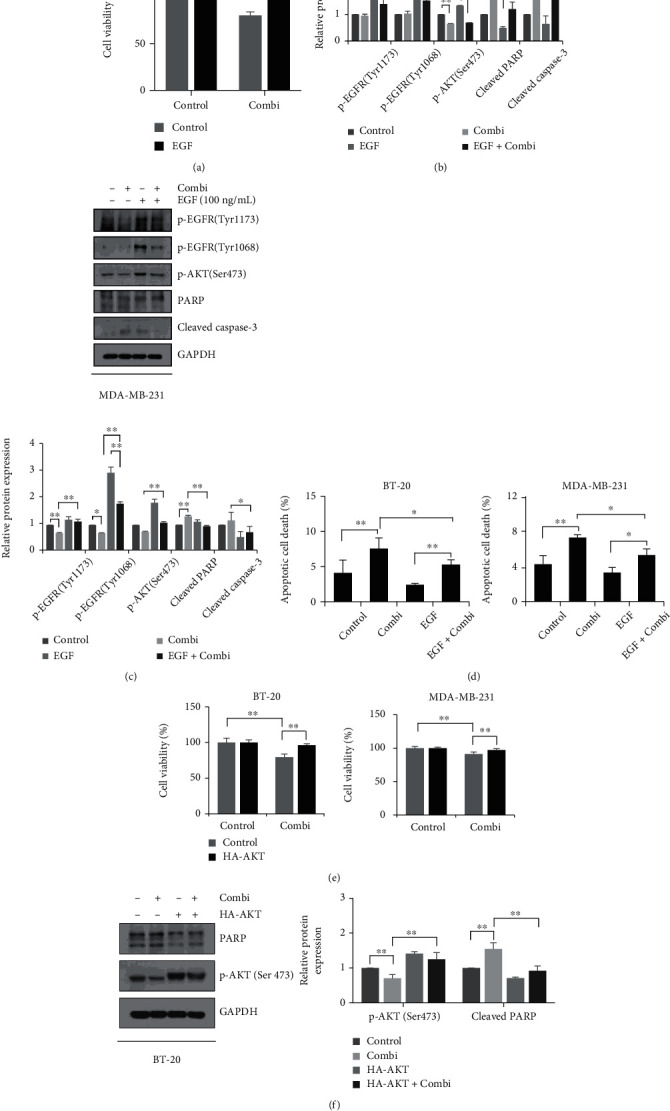
Combination treatment induces the apoptotic cell death through EGFR-AKT signaling suppression. Cells were pretreated with EGF (100 ng/mL) in serum-free media for 15 min and then treated with SH003 (100 *μ*g/mL) and DTX (10 nM or 100 nM) for 24 h. (a) Cell viability was measured by MTT assay. (b, c) Cells were harvested with 2× sample buffer and indicated proteins were analyzed by western blotting. The bar graph indicates the relative protein expression and normalized to GAPDH. (d) After drug treatment for 24 h, cells were harvested and stained with Annexin V/7-AAD. Apoptosis was analyzed by flow cytometry. (e) Cells were transfected with pCDNA3-Myr-HA-AKT2 plasmid for 24 h. And then cells were treated with SH003 and DTX for 24 h. Cell viability was analyzed using MTT assay. (f, g) Apoptosis marker and the expression of AKT phosphorylation were detected by western blotting. The results represent mean ± SD on three independent experiments. ∗*P* < 0.05, ∗∗*P* < 0.01.

Next, we examined whether inhibited EGFR-AKT signaling pathway is directly related to cell death mechanism by combinatorial treatment. Upon epidermal growth factor (EGF) binds to EGFR, it activates AKT-mediated cancer cell proliferation and metastasis. As shown in Figure 4(a), the effect of combination was blocked by EGF treatment in two breast cancer cells. EGF treatment abolished the EGFR and AKT inactivation by combination treatment, but also increased apoptosis markers were inhibited by EGF (Figures 4(b) and 4(c)). Also, EGF rescued both BT-20 and MDA-MB-231 cells from the apoptosis induction by SH003 and DTX (Figure 4(d)), suggesting the inhibition of EGFR signaling involved in apoptotic cell death and AKT inhibition by the combined treatment. We then found the effect of downstream AKT on breast cancer cell death. In Figure 4(e), when AKT was overexpressed, the combination of SH003 and DTX increased the cell viability compared to the control group in BT-20 and MDA-MB-231 cells. In addition, AKT-overexpressed cells prevented the cleavage of PARP, an apoptotic marker, under combination treatment (Figures 4(f) and 4(g)). Based on these results, we suggest that EGFR-AKT pathway is likely to be the main targets of SH003-DTX combination treatment on breast cancer.

### 3.4. Combination with SH003 and DTX Suppresses the Tumor Growth and EGFR Phosphorylation *In Vivo*

**Figure 5:**
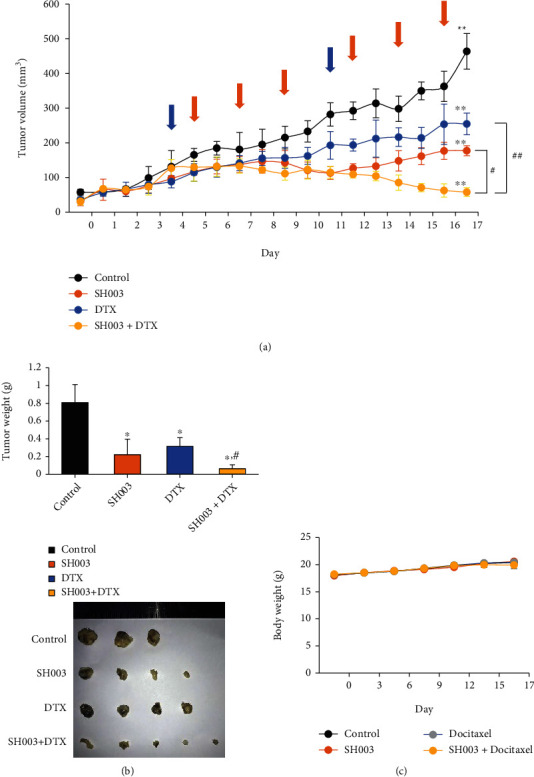
SH003 in combination with DTX enhances the tumor suppression in BT-20 xenograft mouse model. BT-20 cells (1 × 10^7^ cells/100 *μ*L PBS) were inoculated subcutaneously in the right flank of BALB/c nude mice. Mice were divided into four groups; control (*n* = 3), SH003 (*n* = 4), DTX (*n* = 4), and SH003 + DTX (*n* = 5). After tumor size was reached at about 100 mm^3^, the mice were treated with indicated drugs. (a) DTX was administered at dose of 15.28 mg/kg once a week by intravenous injection (blue arrow). SH003 was treated orally at a dose of 557.57 mg/kg 3 times a week (orange arrow). Tumor volumes were measured using a caliper daily. ∗*P* < 0.05 vs. control; ^#^*P* < 0.05 vs. SH003 or DTX by one-way ANOVA with Bonferroni's post hoc test. (b) The graph shows tumor weight. ∗*P* < 0.05, ∗∗*P* < 0.01 vs. control; ^#^*P* < 0.05, ^##^*P* < 0.01 vs. DTX by one-way ANOVA followed by Dunnett's test. (c) Body weight was measured twice a week. Data were expressed as the mean ± SD.

To investigate the efficacy of SH003 combined with DTX *in vivo*, we observed antitumor effect in BT-20 breast cancer xenograft mouse model. Combinatorial treatment of SH003 (557.57 mg/kg) and DTX (15.28 mg/kg) most effectively suppressed tumor growth (Figure 5(a)). Consistently, the combinatorial treatment reduced tumor weight and size (Figure 5(b)). However, those drugs did not affect body weight (Figure 5(c)).

**Figure 6:**
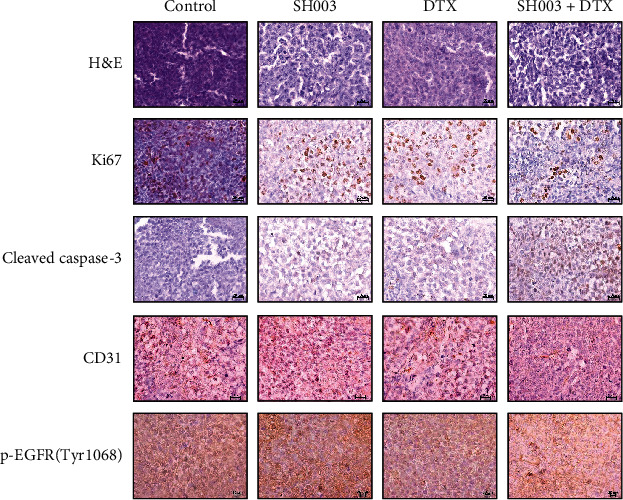
Histological analysis of BT-20 xenograft mouse model. Tumor tissues were stained with hematoxylin & eosin (H&E) and immunostained with Ki67, cleaved caspase-3, CD31, and p-EGFR (Tyr 1068). Scale bar indicates 20 *μ*m.

Our immunohistochemistry confirmed that the combinatorial treatment effectively blocked tumor growth, as it reduced Ki67-positive cell numbers but increased cleaved caspase-3-positive cell numbers (Figure 6, second and third rows). Accordingly, the combinatorial treatment reduced CD31-positive cell numbers (Figure 6, the fourth row). Moreover, the combinatorial treatment reduced the number of cells expressing EGFR phosphorylation at Tyr 1068 (Figure 6, the fifth row).

## 4. Discussion

**Figure 7:**
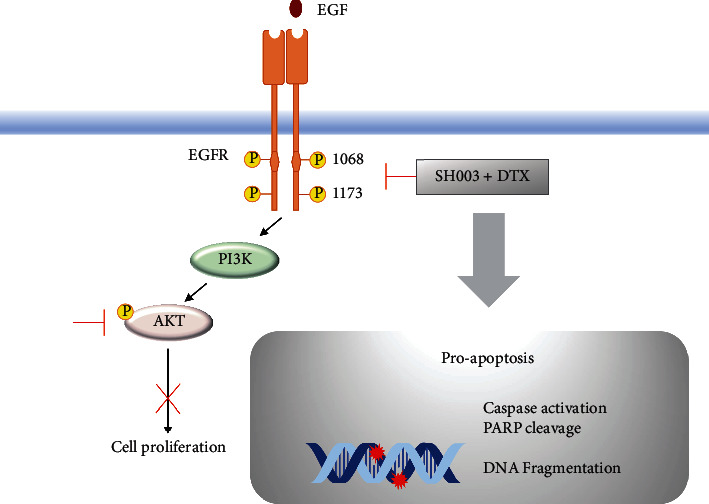
Schematic diagram of regulation mechanism by combined with SH003 and DTX on TNBC.

Although DTX has been used in treatment of many cancer patients, high dose of DTX induces severe side effects including anemia, peripheral neuropathy, and nausea. Moreover, low sensitivity and acquired drug resistance of DTX are still problems [38, 39]. For enhancing the effectiveness of DTX, studies about the combination therapy have been investigated in clinical models, especially in TNBC patients [40, 41]. In this study, we evaluated the synergistic effect of SH003 and DTX in TNBC cell lines and found that SH003 enhances the anticancer effect of DTX by inducing apoptosis and inhibiting the EGFR-AKT signaling pathway of TNBC *in vitro* and *in vivo* with no adverse effect (Figure 7).

In combination therapeutics with synthetic drugs, role of natural products is highly significant due either to a promotion in bioavailability and to a reduction of drug dose *via* synergistic effect [42]. Natural agents such as curcumin, 20S-protopanaxadiol, and flavonoids showed the combination effect with anticancer drugs by inhibiting cell proliferation or drug resistance mechanisms in breast cancer cells [43–45]. We have reported that SH003 has the anticancer effect and also synergistic effect with chemotherapy in TNBC [20, 24, 26]. Thus, the present study is aimed at determining the highest effective dose of SH003 and DTX which has the synergistic effect against TNBC.

Combination therapy is the result of drug interactions, and it is important to find effective drug pairs. In particular, response rates to anticancer drugs in cancer cells could be different because of cancer heterogeneous characteristics, so an appropriate target dose of each drug in combination regimen should be determined. In addition, in order to apply to patients, it is necessary to determine the optimal combination dose through clinical trials [46]. Our results indicated the drug synergism at lower concentrations of SH003 and DTX in vitro, but this synergistic effect did not increase at higher concentrations of SH003. In particular, high dose of SH003 showed antagonistic action in BT-20 cells. But we observed that the combination therapy had antitumor efficacy at the dose used in clinical trial. By using the concentrations of DTX (75 mg/m2) and SH003 (4800 mg/65 kg) determined by MTD and NOAEL studies, we validated the tumor suppression effect of combination treatment, minimizing the side effects in BT-20 cells. These results suggest that clinical studies should be conducted to find an appropriate dose combination regimen that works synergistically while reducing possible side effects. Thus, we demonstrated that SH003 can be a potential agent for combinatorial therapy with DTX, expecting to minimize DTX toxicity in clinical use.

Because high level of EGFR is discovered in about 40% of TNBC, EGFR is a promising target for treatment of TNBC. EGFR-targeted therapy has been developed and used to study for TNBC treatment. Currently, clinical trials of DTX combined with anti-EGFR antibody are ongoing in TNBC patients (NCT01939054) [47]. Although many clinical trials have attempted EGFR-targeting therapies in patient with TNBC subtype, most patients indicated no significant differences in overall response rate [6]. In case of NSCLC treatments, wild-type EGFR NSCLC patients also have low response to EGFR-TKI (tyrosine kinase inhibitors) treatment due to EGFR-TKIs are mostly effective in NSCLC harboring EGFR activating mutations [48]. These results suggest that the development of novel therapeutics targeting wild-type EGFR can overcome failure of EGFR-TKI therapy in TNBC [49]. Our study demonstrated that combinatorial treatment of SH003 and DTX significantly suppresses phosphorylation of EGFR *in vitro* and *in vivo* model. Thus, we suggest that this combinatorial treatment may be an effective therapy for wild-type EGFR amplified TNBC. Further studies are needed to confirm if SH003 and DTX treatment improves EGFR-TKI sensitivity in wild-type EGFR TNBC.

Many studies have shown that the dysregulation of EGFR signaling pathways including AKT, ERK and STAT3 is associated with tumor proliferation and survival [50–52]. Oncogenic function of these pathways contributes to chemoresistance and metastasis in TNBC and thus has been regarded as potential therapeutic targets [37, 53, 54]. We demonstrated that the combinatorial treatment induces apoptotic cell death by suppressing the activation of AKT in both BT-20 and MDA-MB-231 cells. SH003 combined with DTX significantly inhibited ERK and STAT3 phosphorylation in BT-20 cells while activity of ERK and STAT3 is not changed in MDA-MB-231 cells. In the recent study, it was reported that ERK is constitutively activated in MDA-MB-231 cells when compared with BT-20 cells [55]. Also, the expression of STAT3 protein was similar in BT-20 and MDA-MB-231 cells, but a level of STAT3 phosphorylation showed relatively low activity in MDA-MB-231 cells. These diversities of STAT3 activity can be caused by protein expression of ErbB family members as STAT3 upstream pathway [56]. In the therapy of cancer patients through EGFR inhibition, it has been reported that the regulation of the EGFR downstream proteins is important to determine the clinical response [57–59]. Taken together, the different response to combinatorial treatment in both cell lines can be explained by cell line-specific genetic characters. These results can support our finding that the combinatorial treatment more effectively inhibits the proliferation of BT-20 cells at low dose compared with MDA-MB-231 cells. In addition, it can be considered as a biomarker to determine the efficacy and reactivity of drugs depending on which EGFR downstream signaling is regulated.

## 5. Conclusions

The present study suggests that DTX in combination with SH003 would be a good strategy to treat TNBC patients. Furthermore, this combination reduced the EGFR activity and its downstream AKT signaling pathway as a major treatment target for EGFR-amplified TNBC. According to these observations, we also suggest that combination treatment of SH003 and DTX is possible to be considered as an effective drug combination strategy for the treatment of patients with TNBC.

## Figures and Tables

**Table 1:** Combination index value of SH003 and DTX in BT-20 cells.

Drug		BT-20
DTX (nM)	SH003 (*μ*g/mL)	Combination index (CI)
1	100	0.44311
	300	0.70508
	500	1.14198
10	100	0.23670
	300	0.70086
	500	1.14927
100	100	0.35019
	300	1.05284
	500	1.57479
1000	100	1.12051
	300	4.74933
	500	10.8015

**Table 2:** Combination index value of SH003 and DTX in MDA-MB-231 cells.

Drug		MDA-MB-231
DTX (nM)	SH003 (*μ*g/mL)	Combination index (CI)
1	100	1.85E20
	300	5.90E23
	500	7.01930
10	100	3.82112
	300	3.16236
	500	1.17233
100	100	0.09965
	300	0.27457
	500	0.42340
1000	100	0.19020
	300	0.45114
	500	0.57037

## Data Availability

No data were used to support this study.

## Abbreviations

TNBC: Triple-negative breast cancer
DTX: Docetaxel
CI: Combination index
Am: *Astragalus membranaceus*
Ag: *Angelica gigas*
Tk: *Trichosanthes Kirilowii Maximowicz*
EGFR: Epidermal growth factor receptor
TKI: Tyrosine kinase inhibitor.
